# Brassinolide Enhances the Level of Brassinosteroids, Protein, Pigments, and Monosaccharides in *Wolffia arrhiza* Treated with Brassinazole

**DOI:** 10.3390/plants10071311

**Published:** 2021-06-28

**Authors:** Magdalena Chmur, Andrzej Bajguz

**Affiliations:** Department of Biology and Plant Ecology, Faculty of Biology, University of Bialystok, Ciolkowskiego 1J, 15-245 Bialystok, Poland; m.chmur@uwb.edu.pl

**Keywords:** biosynthesis inhibitor, duckweed, occurrence, overcome, phytohormones

## Abstract

Brassinolide (BL) represents brassinosteroids (BRs)—a group of phytohormones that are essential for plant growth and development. Brassinazole (Brz) is as a synthetic inhibitor of BRs’ biosynthesis. In the present study, the responses of *Wolffia arrhiza* to the treatment with BL, Brz, and the combination of BL with Brz were analyzed. The analysis of BRs and Brz was performed using LC-MS/MS. The photosynthetic pigments (chlorophylls, carotenes, and xanthophylls) levels were determined using HPLC, but protein and monosaccharides level using spectrophotometric methods. The obtained results indicated that BL and Brz influence *W. arrhiza* cultures in a concentration-dependent manner. The most stimulatory effects on the growth, level of BRs (BL, 24-epibrassinolide, 28-homobrassinolide, 28-norbrassinolide, catasterone, castasterone, 24-epicastasterone, typhasterol, and 6-deoxytyphasterol), and the content of pigments, protein, and monosaccharides, were observed in plants treated with 0.1 µM BL. Whereas the application of 1 µM and 10 µM Brz caused a significant decrease in duckweed weight and level of targeted compounds. Application of BL caused the mitigation of the Brz inhibitory effect and enhanced the BR level in duckweed treated with Brz. The level of BRs was reported for the first time in duckweed treated with BL and/or Brz.

## 1. Introduction

Brassinosteroids (BRs) are a class of steroid phytohormones represented by the above 70 compounds as free compounds or fatty acid and glucose conjugates. They are widely distributed in plants; their presence has been confirmed in algae, mosses, and vascular plants [1,2,3,4,5]. Brassinolide (BL) is the first discovered, simultaneously the most widespread and active representative of BRs [5,6]. BRs regulate many physiological processes in plants, including cell division and elongation, vessel differentiation, reproductive development, seed germination, flowering, pollen development, modulation of gene expression, maturation, and aging of the plant. Moreover, BRs significantly improve the efficiency of transpiration and cause the increase of chlorophylls, carbohydrates, and protein contents [7,8,9,10,11]. Besides, BRs participate in plants’ tolerance to various abiotic stresses, such as hypoxia, heavy metals, drought, salinity, and oxidative stress. Moreover, BRs are involved in plant protection against pathogen attacks [12,13,14,15].

Depending on the number of carbon atoms in a molecule, these sterols are divided into C_27_-,C_28_- or C_29_-type of BRs, among which, the most widespread are C_28_-BRs [5]. Their biosynthesis occurs through the early or late C6 oxidation pathway with a precursory compound named campestanol (CN). During the late C6 pathway, CN is hydroxylated in C-22 position into the 6-deoxocatasterone (6dCT), which is hydroxylated in C-23 position into the 6-deoxoteasterone (6dTE). The resulting compound is converted into the 3-dehydro-6-dTE, which is converted to 6-deoxotyphasterol (6dTY). Then, 6-deoxoTY is hydroxylated to 6-deoxocastasterone (6dCS), which is converted to castasterone (CS). In contrast, the early C6 oxidation pathway begins from the oxidation of CN to 6-oxoCN. Then, 6-oxoCN is converted to catasterone (CT), teasterone (TE), 3-dehydroteasterone, typhasterol (TY), CS, and BL, respectively [3,16,17] BRs’ biosynthesis can be blocked through the application of specific inhibitors [18]. One of them is brassinazole (Brz), a synthetic triazole-type inhibitor of BRs’ synthesis, which blocks the hydroxylation of CN to the 6dCT and hydroxylation of 6-deoxoCT to the 6dTE during the late C6 pathway, and analogously, it inhibits the conversion of 6-oxocampestanol (6-oxoCN) to CT and conversion of CT to the TE during the early C6 pathway [19,20]. Plants treated with Brz indicate the phenotype changes, primarily manifested in dwarfism or growth inhibition. However, exogenously applied BRs can alleviate these unfavorable morphological alterations. Brz application aims at confirmation of BRs’ biological activity and role in plants [21,22,23,24,25,26]. Another type of BR biosynthesis inhibitor is Brz2001, a modified form of Brz containing an allyl moiety instead of the methyl group [16,18].

The duckweeds belonging to the *Wolffia* genus are the smallest angiosperms with strongly reduced organs; they do not create a stem, leaves, and roots system. The body size of *W. arrhiza* reaches about 1 mm in diameter. Rarely does flowering occur; therefore, most commonly, it reproduces vegetatively. Despite a simple body structure, *W. arrhiza* has an excellent adaptation for living in the aquatic environment. In organic-rich water, it changes the way of feeding on photoautotrophic into mixotrophic or heterotrophic. Moreover, *W. arrhiza* can accumulate xenobiotics, e.g., metals, radionuclides, nanoparticles, pesticides, and pharmaceuticals, from polluted water. Therefore, the fast reproductions, good absorption ability of organic compounds, and simple breeding methods of *W. arrhiza* provide this duckweed to be a good plant for practical usage in wastewater treatment [27,28,29,30].

The present work is a continuation of the research performed by Bajguz and Asami [31], in which the growth and level of chlorophylls, carotenoids, monosaccharides, and proteins under the influence of EBL and/or Brz2001 in *W. arrhiza* cultures was studied. The present study’s main aim was to determine the endogenous level of Brz and BRs in the duckweed treated with BL and/or Brz. Besides hormone analysis, the duckweed growth rate and level of photosynthetic pigments, proteins, and monosaccharides under the influence of Brz and BL were also analyzed. For this, the following hypotheses were tested: (1) BL has a stimulatory effect on the growth and endogenous level of BRs, photosynthetic pigments, proteins, and monosaccharides in *W. arrhiza*; (2) Brz decreases the fresh weight and content of BRs and primary metabolites in the duckweed; (3) Brz is absorbed by *W. arrhiza* cultures; (4) BL effectively overcomes the repressive impact of Brz on the growth rate and level of targeted compounds; (5) the effect of BL and Brz in *W. arrhiza* differs in a concentration-dependent manner. LC-MS/MS system was used to identify and quantify BRs as well as Brz. The LC-MS/MS with a multiple reaction monitoring (MRM) mode is one of the most predominant analytical methods of BRs’ analysis due to the high sensitivity and efficient separation performance [5,32]. For the analysis of pigments belonging to the chlorophylls, carotenes, and xanthophylls, the HPLC method was used. Obtained results show the presence and essential role of BRs in *W. arrhiza* growth.

## 2. Results

### 2.1. Growth Rate of W. arrhiza

**Figure 1:**
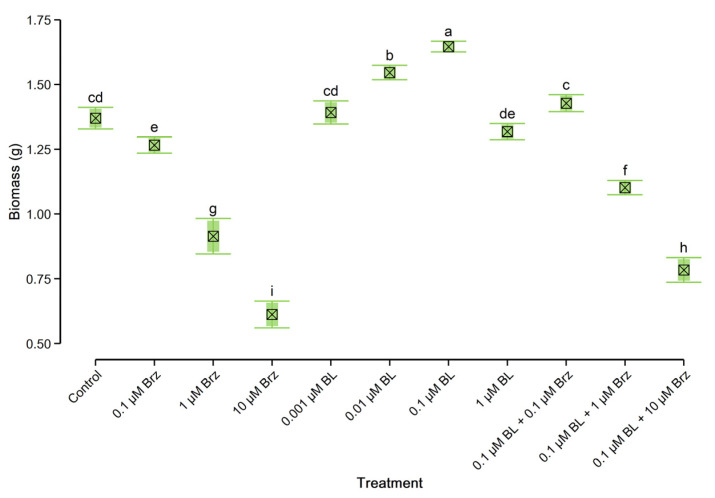
The effect of BL and/or Brz on *W. arrhiza* biomass after 7 days of cultivation. The crossed square shows the mean (*n* = 5). The lower and upper hinges correspond to the lower and upper bounds of 95% confidence interval of the mean. The lower and upper whiskers extend from the hinge to mean ± standard deviation. Means with the same letters are not significantly different (*p* ≥ 0.05) according to Tukey’s post-hoc test.

The effect of BL, Brz, and the combination of BL with Brz on the *W. arrhiza* growth rate is presented in Figure 1. The initial weight of duckweed in each variant was 1 g. After 7 days of cultivation, the biomass of untreated plants increased up to 1.37 g. Application of BL in range of concentration 0.001–0.1 µM caused the promotive effect on *W. arrhiza* growth. The slight increases were observed in plants treated with 0.001 µM BL, while the most stimulatory effect was noted in plants exposed to the 0.1 µM BL. The cultivation with the addition of 0.1 µM BL caused a considerable increase of duckweed weight, up to 1.65 g, and this is a 20% rise compared to the control plants. Whereas treatment with 1 µM BL had a slight inhibitory effect on the growth rate, causing a 4% decline of biomass in relation to the untreated plant.

An opposite effect on duckweed growth was observed in plants treated with BR biosynthesis inhibitor. Exogenously applied Brz led to the decrease of *W. arrhiza* weight in a concentration-dependent manner. Thus, the application of 0.1 µM Brz had a slight inhibitory effect on plant growth, whereas under the influence of 1–10 µM Brz, the fresh weight of duckweed was considerably reduced, reaching the highest of a 55% decrease in plant treated with 10 µM Brz.

The inhibitory effect of Brz on *W. arrhiza* growth was reversed through the application of 0.1 µM BL. Combination of 0.1 µM BL with 0.1 µM Brz caused a statistically inconsiderable enhance of plant weight compared to the control. The mixtures of 0.1 µM BL with 1–10 µM Brz also positively affected duckweed weight, and caused the 20% and 28% increases to plant treated with 1–10 µM Brz alone, respectively. However, the biomass values of duckweed exposed on 0.1 µM BL with 1–10 µM Brz was lower than the control group, by 20% and 43%, respectively.

### 2.2. Brassinazole and Brassinosteroids’ Content in W. arrhiza

Table 1 presents the endogenous level of BRs and Brz in *W. arrhiza* treated with BL and/or Brz. The LC-MS/MS analysis indicated the occurrence of nine BRs, i.e., BL, 24-epibrassinolide (EBL), 28-homobrassinolide (HBL), 28-norbrassinolide (norBL), CT, CS, 24-epicastasterone (ECS), TY, and 6dTY.

In untreated plants, the highest content of CS, CT, and BL was noted (2.77 ng/g, 1.96 ng/g, and 1.79 ng/g, respectively). Simultaneously, the lowest amount of ECS and 6dTY was observed (0.03 ng/g for both of them). The total content of BRs in the control group was 7.68 ng/g. Furthermore, the effect of treatment with BL, Brz, and mixture of BL with Brz on BRs’ content was studied. Application of 0.001–0.1 µM BL had a stimulatory effect on the content of all detected BRs. The addition of 0.001 µM BL caused a slight increase of BRs level, whereas exposition on 0.01–1 µM BL led to the significant enhancement of BRs’ content in *W. arrhiza*. For instance, the endogenous CS and CT level increased sequentially by 70% and 45% in plants treated with 0.1 µM BL to the control. Additionally, the endogenous level of BL was considerably higher in plant exposed on BL, that is related with the absorption of the exogenously applied hormone by *W. arrhiza*. Furthermore, the content of EBL also was significantly higher in plants treated with BL. The amount of BL and EBL enhanced gradually with the increasing concentrations of BL. Whereas the overall content of BRs, except BL and EBL, was 10.38 ng/g in plant exposed on 0.1 µM BL. Thus, this is an almost 2-fold increase of BRs’ content to the control group, in which total BRs’ content, without BL and EBL, was 5.72 ng/g. In duckweed exposed on 1 µM BL, the overall content of HBL, norBL, CT, CS, ECS, TY, and 6dTY was 6.67 ng/g. To summarize, the treatment with 0.1 µM BL had the most stimulating effect on the content of BRs in *W. arrhiza*.

An opposite effect was observed in a group of plants treated with 0.1–10 µM Brz. The endogenous level of BRs decreased proportionally to the increase of Brz concentration. In duckweed exposed on 10 µM Brz, TY and 6dTY were not detected, whereas the BL level decreased by 92% in the untreated plants. The total BRs’ content was 1.49 ng/g, 81% lower than in control. The strong inhibitory action is probably connected with a huge absorption of Brz from the medium. In the plants exposed to 1 µM Brz, the endogenous inhibitor level was about 7000 ng/g. Therefore, 11% of Brz was absorbed by 1 g of duckweed.

The inhibitory effect of Brz on the BRs’ synthesis was reduced by the application of 0.1 µM BL. In plants treated with the combination of 0.1 µM BL with 0.1 µM Brz, the level of BRs was slightly higher than in control. The application of 0.1 µM BL with 1 µM Brz caused a significant increase of BRs’ content to plant treated with 1 µM Brz alone, except for ECS and TY. However, the amount of BRs was lower by 40% compared to the control, while BL and EBL levels were lower than in plants treated with BL alone. The least restored effect was observed in duckweed treated with a mixture of 0.1 µM BL with 10 µM Brz. The total inhibition of TY and 6dTY synthesis under the influence of 10 µM of the addition of BL did not reverse Brz. Whereas the endogenous level of BL and EBL was considerably lower than the plant treated with BL alone. The content of remaining BRs was slightly higher than in plant exposed only with 10 µM Brz. For example, the content of CS increased from 0.73 to 0.87 ng/g, and the level of CT enhanced from 0.52 to 0.7 ng/g. However, in plants exposed to 0.1 µM BL with 10 µM Brz, the endogenous level of BRs was three times lower compared to the control. The addition of µM 0.1 BL also resulted in a reduction of Brz accumulation in duckweed treated with 0.1 µM and 1 µM Brz; however, in the plant exposed with 10 µM Brz, the endogenous level of inhibitor was slightly higher compared with plant treated with 10 µM Brz alone.

### 2.3. Photosynthetic Pigments’ Content in W. arrhiza

The level of photosynthetic pigments in duckweed *W. arrhiza* exposed on Brz and/or BL is shown in Table 2. In the control group, the total content of chlorophylls, carotenes, and xanthophylls was 204.35, 3.07, and 9.89 ng/g, respectively. The treatment with BL enhanced the pigment synthesis in a concentration-dependent manner. Application of 0.001 µM BL caused an inconsiderable increase in the level of chlorophylls, carotenes and xanthophylls compared with untreated duckweed. The treatment with 0.1 µM BL had the most stimulatory effect on pigment content. The endogenous level of chlorophyll *a* and *b* increased by 39% in the plant treated with 0.1 µM BL to the control. Similarly, carotenoids’ content increased by 69% and 48%, respectively, compared with the plant without hormone addition.

The application of Brz resulted in a decrease of synthesis in all detected pigments. The overall pigment content decreased proportionally with the increasing inhibitor concentration, reaching the most inhibitory effect in duckweed treated with 10 µM Brz. For example, the level of chlorophyll *a* in a plant with 10 µM Brz was 54% lower than in unexposed plants. The treatment with 0.1 µM BL mitigated the inhibitory effect of Brz on pigment synthesis. In duckweed exposed on simultaneous action of 0.1 µM BL and 0.1 µM Brz, their overall content was 20% higher than in the control group. Whereas the combinatory effect of 0.1 µM BL with 1 µM Brz or 10 µM Brz resulted in a decline of pigment content to the untreated plants, but their level was higher than in plants treated with 1 µM Brz and 10 µM alone.

### 2.4. Protein and Monosaccharides’ Content in W. arrhiza

**Figure 2:**
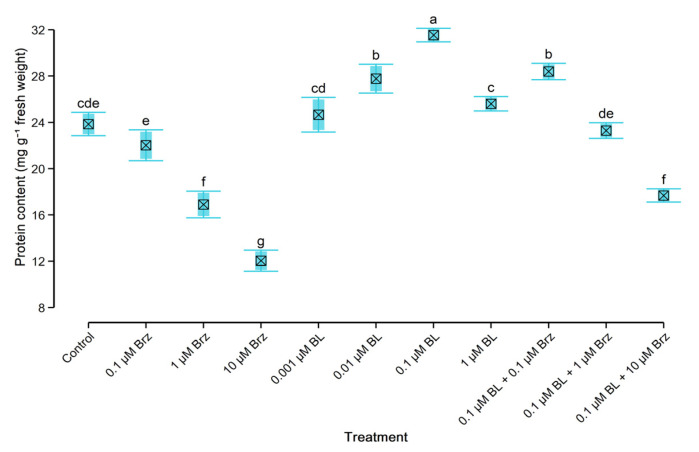
The effect of BL and/or Brz on protein content in *W. arrhiza* after 7 days of cultivation. The crossed square shows the mean (*n* = 5). The lower and upper hinges correspond to the lower and upper bounds of 95% confidence interval of the mean. The lower and upper whiskers extend from the hinge to mean ± standard deviation. Means with the same letters are not significantly different (*p* ≥ 0.05) according to Tukey’s post hoc test.

**Figure 3:**
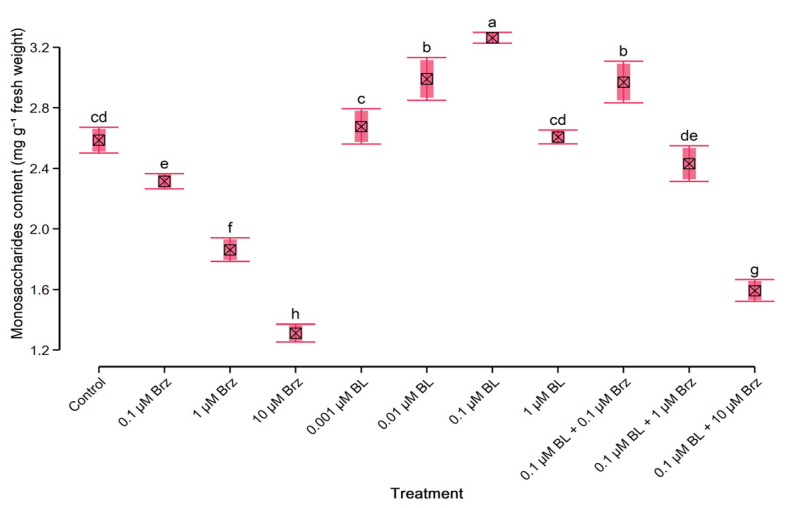
The effect of BL and/or Brz on content of monosaccharides in *W. arrhiza* after 7 days of cultivation. The crossed square shows the mean (*n* = 5). The lower and upper hinges correspond to the lower and upper bounds of 95% confidence interval of the mean. The lower and upper whiskers extend from the hinge to mean ± standard deviation. Means with the same letters are not significantly different (*p* ≥ 0.05) according to Tukey’s post hoc test.

Application of BL at the range of concentrations of 0.01–1 µM had a stimulatory effect on the protein (Figure 2) and monosaccharides’ (Figure 3) content. The greatest increase of these compounds was observed under the influence of 0.1 µM BL. Thus, the total content of protein enhanced by 25%, and the total level of monosaccharides increased by 22% to the untreated plant.

In opposition to the BL, the treatment with inhibitor caused a considerable decrease in the contents of protein (Figure 2) and monosaccharides (Figure 3). Plant exposition on 0.1 µM Brz had a slight inhibitory effect on amount of monosaccharides while protein did not change significantly. However, in duckweed exposed to the 10 µM Brz, the level of protein and monosaccharides decreased by 50% and 52% compared to the control, respectively. Moreover, the effect of 0.1 µM Brz on the protein level and monosaccharides was suppressed by the application of 0.1 µM BL. The use of 0.1 µM BL with 0.1 µM Brz led to the 15% increase of the amount of protein and monosaccharides to the control. Whereas the amount of these compounds in duckweed treated with 0.1 µM BL with 1 µM Brz, or 10 µM Brz was lower than in control but higher than in the group treated with 1 µM Brz or 10 µM Brz alone.

## 3. Discussion

### 3.1. Exogenous Brassinolide Improves the Growth Rate and Content of Endogenous Hormones and Primary Metabolites

Phytohormones promote plant growth and biosynthesis of targeted compounds in a concentration-dependent manner. An activity of exogenously applied hormones depends on their absorption efficiency by plants. Exogenous BRs are uptaken and transported in dependence on the application method. In plants, the most efficient absorption of BRs occurs by the roots due to their function which is to uptake substances from the soil [33]. While in water, deprived of stem and roots of plants, absorption of BRs and other molecules takes place through the endocytosis process [29]. As one of the most active BRs, BL plays a vital role in plant growth and regulation of physiological processes [34,35]. In the present study, the enhancement of growth rate and level of detected BRs in the duckweed *W. arrhiza* exposed on BL was observed. Moreover, the content of primary metabolites, such as photosynthetic pigments, monosaccharides, and protein, increased in the presence of BL.

The various hormone concentrations may have opposite effects on plant growth and metabolism. BL in the range of 0.001–0.1 µM had the stimulatory effect on the *W. arrhiza* growth; however, treatment with 1 µM BL caused a slight decrease of duckweed growth. Plant growth is dependent upon the synthesis of nucleic acids and protein. It was demonstrated that the activation of the growth of plant tissue and higher levels of DNA and RNA polymerase is manifested by the increase of the content of nucleic acids and protein. It may be concerned directly or indirectly, with growth promotion induced by BRs that enhancement of DNA and RNA polymerase activities may be a result of regulation of gene expression. Moreover, the high doses of BRs may act antagonistically—in this case, the rate of growth is decreased. Suppression of plant growth is a result of mitosis blocking, DNA replication inhibition, the breakdown of cell integrity and cell membrane, and the degradation of cell wall polysaccharides induced by high doses of BRs [36,37,38,39]. Jiroutova et al. [40] showed that high EBL concentration (above 0.5 µM) considerably enhanced ethylene production, which induces plant senescence. Furthermore, high doses of BRs cause gibberellin inactivation, which is correlated with the inhibition of rice (*Oryza sativa*) cell elongation [41]. Other studies demonstrated the stimulatory effect of BRs; however, the growth rate under the influence of various BR concentrations differed among plant species. For instance, in the green alga *Acutodesmus obliquus*, the most promotive effect on the number of algal cells was reached at a concentration of 1 µM EBL [42], while the greatest increases of the *Chlorella vulgaris* growth was observed in alga treated with 0.01 µM BL [43]. In another study, the most promotive effect on the weight of *Brassica junceae* shoots and roots was noted after treatment with 0.5 µM BL [44]. The present study shows that 0.1 µM BL stimulated duckweed growth the most. Summarizing, the explanation of interactions between BRs and other phytohormones on plant growth and development requires further analysis.

The most important part of the presented work was confirmation of the occurrence and determination of the endogenous level of BRs under the influence of exogenously applied BL with Brz in duckweed *W. arrhiza*. Although the presence of BRs has been evidenced both in angiosperms and gymnosperms plants, *W. arrhiza* remains the only species among Lemnoideae in which these hormones were identified [27]. In our study, the content of BRs is in the range of 0.03–2.77 ng/g in the control group of duckweed. The presence of BRs belonging to the early and late C-6 oxidation pathway was reported. In addition, the occurrence of norBL, which possesses 27 atoms of carbon, and HBL with 29 carbon atoms was also identified. Among all detected BRs, CS, CT, and BL dominate, while ECS and 6dTY occur in a trace amount. In turn, the presence of 6dCT was not detected. These results suggest that the early C-6 oxidation pathway is predominant. Additionally, the presence of EBL and norBL in *W. arrhiza* was noted for the first time. In other reports, the levels and the profiles of BRs varied in dependence on family, species, or environment. Among freshwater plants, eight various BRs were detected in *A. obliquus* [45], while the presence of the most active BL and CS was confirmed in many algal strains [46,47]. Whereas, among freshwater angiosperms, the level of BRs was not studied.

The duckweed treatment with BL had a positive effect on the biosynthesis of all detected BRs. Similarly to the growth rate, the most increase of BRs’ level was reported under the influence of 0.1 µM BL, while during the treatment with 1 µM BL, the content of BRs declined. These BRs’ level alterations may be associated with the direct effect of exogenously applied BRs on their biosynthetic pathways [48]. Furthermore, the application of BL to hydroponically cultured duckweed influenced the considerable absorption of BL manifested in the increases of its endogenous level. Moreover, the endogenous level of EBL was considerably enhanced in duckweed treated with BL. It suggests that in abundant conditions of BL, BL can be converted directly into EBL. These results are similar to the studies of Janeczko and Swaczynova [49], who demonstrated the increases of endogenous content of BL and CS in wheat (*Triticum aestivum*) seedlings treated with 0.1 µM EBL. The stimulatory effect of 0.1 µM EBL on the level of BRs in barley (*Hordeum vulgare*) was also reported [48].

As is known, chlorophylls are the necessary photosynthetic pigments, and their major role is light-absorbing from solar power for ATP synthesis. In plants, two types of chlorophylls are presented, predominantly chlorophyll *a* and, in less amount, chlorophyll *b*. Phytohormones and environmental conditions determine their biosynthesis and degradation. Determination of the chlorophyll content is used to analyze the photosynthesis rate in plants. As the second group of photosynthetic pigments, carotenoids possess antioxidant activity and protect chlorophylls against photo-oxidative destruction [8]. Carotenoids are divided into carotenes, and their oxygen derivatives mean xanthophylls. Whereas xanthophylls are divided into the oxygen-poor compounds containing one or two atoms of oxygen in the molecule, e.g., citranaxanthin, zeaxanthin, lutein, rhodoxanthin, canthaxanthin, cryptoxanthin, and oxygen-rich molecules containing 3 or 4 oxygen atoms, e.g., astaxanthin, antheraxanthin, neoxanthin, flavaxanthin, and violaxanthin [50,51]. Both chlorophylls and carotenoids are essential to the light phase of photosynthesis [52]. The presence of photosynthetic pigments with the specific individual xanthophyll was confirmed mainly in algae [42,53] and useless agricultural plants, e.g., maize (*Zea mays*) [54], wheat [55], or soybean (*Glycine max*) [56]. While in the present studies, the occurrence of two chlorophylls and eight carotenoids was also confirmed (Table 2). The increased level of pigments in duckweed treated with BL shows that exogenously, BL contributes to photosynthesis efficiency improvements. The direct function of BRs in photosynthesis is the increase of plants’ light-capturing efficiency and induction of the activity of chlorophyll biosynthesis enzymes [8]. Referring to the previous studies, EBL caused the increase of pigment content in alga *A. obliquus* [42], *C. vulgaris* [57], and wheat [58]. In another study, Maity and Bera [59] observed the promotive effect of BL on the total chlorophylls’ content in the mung bean (*Vigna radiata*).

Sugars, as signaling molecules, are a great reservoir of carbon and energy necessary for all biochemical changes in plants. Monosaccharides, mainly glucose, are produced during a photosynthesis process [60]. The effective role of BRs in photosynthesis is the improvement of the CO_2_ assimilation in the Calvin cycle, which leads to an increase of Rubisco activity, resulting in enhanced content of monosaccharides [26,31]. Therefore, the rise of monosaccharides’ level in the presence of BL is associated with the increase of photosynthesis activity, resulting from the increases of synthesis of chlorophylls in *W. arrhiza*. These results are in agreement with the literature data. In earlier studies focused on *W. arrhiza*, the level of monosaccharides in the presence of EBL was enhanced [31]. Whereas the analysis of Yu et al. [61] indicated the increase of sugar content in cucumber (*Cucumis savitus*) treated with EBL.

Similar to the sugars, the endogenous content of soluble protein increased in the presence of BL. Moreover, it was evident that the enhanced synthesis of nucleic acids and proteins contributes to the increase of plant growth rate. Treatment with BL causes the acceleration of metabolic processes that favors the accumulation of soluble proteins. Therefore, it suggests that the BL application also affects the level of nucleic acids and, consequently, contributes to the rise in the translation process rate [38,57]. According to the earlier reports, exogenously applied EBL positively affected the protein content in *W. arrhiza* [31]. In another study, the stimulating effect of BL on the level of soluble protein was noted in *V. radiata* [59].

### 3.2. Exogenous Brassinolide Overcame the Negative Effect of Brassinazole

The results presented above show the positive effect of exogenously applied BL on the *W. arrhiza* growth and level of targeted compounds but do not provide certain information about the direct role of endogenous BRs in duckweed development. Therefore, there are two major ways to analyze the functions of endogenous hormones in plants. The first method is to create BRs’ deficient mutants through the mutation of genes encoding key enzymes in BRs’ biosynthesis. In model plants, e.g., *Arabidopsis thaliana*, mutants’ growth is drastically inhibited, the length of hypocotyl is reduced, roots and petioles are shorter, while leaves are smaller and discolored. Moreover, the leaf petiole and blade lengths are significantly reduced, while the stamen filament elongation is impaired, leading to male sterility [62,63,64]. However, the more universal and accessible method is using the BR biosynthesis inhibitors. The plant responses on the treatment with inhibitors led to recognize the importance of BRs in many physiological and biochemical processes in plants. Brz, as a specific inhibitor of BRs, blocks the conversion of 6-oxoCN to CT and conversion of CT to the TE, and analogously, it blocks conversion CN to the 6-deoxoCT and 6-deoxoCT to the 6-deoxoTE. The chemical structure of Brz contains a triazole ring and methyl residue attached to the carbon atom, which includes a hydroxy group. The methyl group is required for the activity of Brz because the compound without these groups does not indicate any inhibiting activity of BRs. The measurement of plant growth rate under the influence of various Brz concentrations was performed. In plants, often with the Brassicaceae family, the hypocotyl elongation in seedlings treated with Brz to untreated plants is compared. The hypocotyl length of cress (*Lepidium sativum*) was considerably lower in cress exposed to 1 µM and 10 µM Brz. Additionally, in plants treated with 0.1 µM Brz, a slight decrease of hypocotyl length was observed [18]. The foliar application of Brz caused a growth decrease in soybean [25]. Besides growth reduction, Brz- treated cress exhibited curled, dark-green leaves [21]. The inhibitory effect of Brz on the hypocotyl elongation in seedlings of barley [48], cucumber, tomato (*Solanum lycopersicum*), and tobacco (*Nicotiana tabacum*) [20], or *A. thaliana* [19] was also reported. Thus, the phenotype modifications of Brz-treated plants are similar to the genetically modified BR-deficient mutants. Whereas in small, deprived-of-the-stem-and-roots freshwater *W. arrhiza*, the growth rate analysis was performed by comparing plant fresh weight. The present study revealed the strong negative effect of 1 µM and 10 µM Brz on duckweed growth. The reproduction of duckweed was inhibited. The weight of cultures exposed on 10 µM Brz was 2-fold reduced after 7 days of treatment. The phenotype of the whole plant was lightened and withered.

Discoloration of duckweed is connected with a considerable decrease of chlorophylls and other pigments. Similar to the pigments, the endogenous amount of monosaccharides and soluble proteins was significantly lower. The decrease of content of monosaccharides, observed in *W. arrhiza* treated with Brz, was caused by the degradation of photosynthetic pigments contributing to decreased photosynthesis rate and monosaccharides’ accumulation. The inhibited growth and reduced concentrations of targeted metabolites are associated with the inhibition of BRs’ biosynthesis. Thus, in the present study, application of Brz resulted in BR-deficiency phenotypes through the decrease of endogenous BR levels. Besides BRs, the endogenous level of Brz in *W. arrhiza* treated with inhibitor was also determined. Interestingly, this is the first report about the content of Brz in duckweed tissues. The previous studies indicated the effect of Brz on the morphological processes in plants but did not provide any data about concentrations of this inhibitor in plant organs. The exogenous treatment with BL reversed the inhibitory effect of Brz on the BRs’ biosynthesis in duckweed. Interestingly, simultaneous application of 0.1 µM BL with 0.1 µM of Brz caused an enhanced level of BRs compared with untreated plants. The co-application of BL with Brz also had a stimulatory effect on the growth and metabolites’ content to the group exposed on Brz alone. Concerning the literature data, exogenous BRs’ influence on their accumulation in plants treated with Brz was exceptionally rarely studied. It was reported that exogenous EBL application overcame the inhibition of BRs’ synthesis caused by treatment with Brz [48]. Thus, the previous research on *W. arrhiza* showed the reduction of fresh weight, proteins, and monosaccharides’ content in cultures treated with Brz2001 and their restoration after application of EBL [31]. Referring to the land plants, BL reversed the dwarf phenotype of *A. thaliana* seedling treated with Brz [20]. While, in plants treated with Brz2001, the recovery effects of treatment with EBL on the length of soybean seedling [65] and algal growth (expressed as a number of cells) [26] were also observed.

## 4. Materials and Methods

### 4.1. Plant Material and Growth Conditions

The culture of *W. arrhiza* (L.) Horkel ex Wimm. was obtained from the Faculty of Biology of the University of Bialystok. The breeding of *W. arrhiza* was conducted under controlled conditions at 22.0 ± 0.5 °C, 16 h photoperiod (photon flux of 100 µmol/m/s), and a relative humidity of 65%. One gram of plant was placed in a sterile, glass vessel containing 200 mL of Hunter medium with the following composition: 500 mg/L EDTA, 500 mg/L MgSO_4_·7H_2_O, 400 mg/L KH_2_PO_4_, 354 mg/L Ca(NO_3_)_2_·4H_2_O, 200 mg/L KOH, 200 mg/L NH_4_VO_3_, 65.9 mg/L ZnSO_4_·7H_2_O, 25.2 mg/L Na_2_MoO_4_ · 2H_2_O, 24.9 mg/L FeSO_4_·7H_2_O, 17.9 mg/L MnCl_2_ 4H_2_O, 14.2 mg/L H_3_BO_3_, 3.95 mg/L CuSO_4_·5 H_2_O, 0.2 mg/L Co(NO_3_)_2_·6H_2_O [66].

The whole experiment was divided into two major steps. In the first stage of the experiment, the cultures of *W. arrhiza* were treated with Brz in the range of concentrations 0.1–10 µM, or BL in the range of concentrations 0.01–1 µM. The varied solutions were prepared through the diluting of hormone and inhibitor in Hunter medium. After 7 days of treatment, the duckweed biomass was separated from the medium by filtration using a vacuum pump (KNF Neuberger, Inc., Trenton, NJ, USA). Then, the collected plant was weighed and homogenized in liquid nitrogen using a mortar and pestle. The obtained powder was used in further analysis. In the second experiment, the cultures of *W. arrhiza* were treated with a mixture of 0.1 µM BL with 0.1, 1, and 10 µM Brz. The concentration of 0.1 M µM BL was selected because it indicated the greatest effect on the growth and the content of analyzed compounds in duckweed. The subsequent parts of the sample preparation were analogs to stage one.

After 7 days of treatment, the analysis of growth and selected biochemical parameters was performed. The measurement of biomass, protein, pigments, and monosaccharides is necessary for each research involving plants because those parameters constitute a basic indicator of plant responses.

### 4.2. Chemicals

The eleven standards of BRs (BL, EBL, HBL, norBL, CS, ECS, 6dCS, CT, 6dCT, TY, and 6dTY, as well as stable isotope-labeled standards of [^2^H_3_]BL and [^2^H_3_]CS, were purchased from OlChemIm (Olomouc, Czech Republic). Brz was purchased from TCI Europe N.V. (Zwijndrecht, Belgium). The initial solution of the inhibitor was prepared by the dissolution of Brz powder in dimethyl sulfoxide (DMSO), while the BL was dissolved in 70% ethanol (EtOH). The final amount of DMSO and EtOH added to the medium did not affect the *W. arrhiza* growth. Bovine albumin standard, all chemicals for Hunter’s medium, Bradford and Somogyi reagents were purchased from Sigma-Aldrich (St. Louis, MO, USA). Methanol (MeOH), EtOH, acetonitrile (ACN), water (LC-MS purity), formic acid (FA), potassium hydroxide (KOH), and 4-(dimethylamino)phenylboronic acid (DMAPBA), and DMSO were purchased from Merck KGaA (Darmstadt, Germany). Ten standards of photosynthesis pigments (chlorophyll *a*, chlorophyll *b*, α-carotene, β-carotene, neoxanthin, violaxanthin, astaxanthin, zeaxanthin, cryptoxanthin, and lutein) were purchased from DHI (Horsholm, Denmark).

### 4.3. Quantification of Brassinazole and Brassinosteroids

The measurement of the endogenous level of Brz and BRs was performed using the LC-MS method. For this purpose, 200 mg of plant powders were placed into 2 mL Eppendorf tubes, suspended in 1 mL 95% (*v*/*v*) MeOH, and homogenized in a bead mill (50 Hz, 10 min, TissueLyser LT; Qiagen GmbH, Düsseldorf, Germany) using three 2 mm tungsten balls. Then, the homogenates were centrifuged (2800× *g*, 10 min; MPW-55 Med. Instruments, Gliwice, Poland), and supernatants were transferred to the glass flasks with a flat bottom. The remaining precipitates were suspended in MeOH and centrifuged again. This procedure was repeated five times. The final volume of supernatants (5 mL) was mixed (90 rpm, Laboratory shaker LC-350, Pol-Eko-Aparatura, Wodzisław Śląski, Poland) in temperature of 5 °C for 12 h. For quantification of BRs, [^2^H_3_]BL (2 ng) and [^2^H_3_]CS (2 ng) were added into the mixture, followed by extraction with MeOH as internal standards. The sample purification was performed according to Xin et al.’s [67] method. Therefore, the obtained supernatant (5 mL) was purified from pigments and other pollutions using solid-phase extraction (SPE) MAX cartridge (6 mL, 500 mg, Waters Corporation, Milford, MA, USA), which was activated and equilibrated with 99.9% MeOH, H_2_O, 1 M KOH, 10% (*v*/*v*) MeOH, and 95% (*v*/*v*) MeOH, respectively. Purified extracts were dried up using a centrifugal vacuum concentrator, reconstructed in 10% (*v*/*v*) MeOH, and passed through Waters SPE MCX cartridge to remove ion contamination. Cartridges were previously activated and equilibrated with 5% (*v*/*v*) FA in 5% (*v*/*v*) MeOH, 5% (*v*/*v*) MeOH, 5% (*v*/*v*) NH_4_OH in 5% (*v*/*v*) MeOH, and 5% (*v*/*v*) MeOH, respectively. Then, samples were eluted using 80% (*v*/*v*) MeOH. Eluents were dried up using a centrifugal vacuum concentrator, suspended in 98 µL of 96% (*v*/*v*) EtOH, and derivatized (45 °C, 1 h), using 2 µL of DMAPBA reagent.

Detection and quantitative analysis of Brz and BR-DMAPBA were performed using the Shimadzu LC-MS-MS-8050 system consisting of pump, degasser, autosampler, column oven, and mass spectrometer with triple quadrupole (Shimadzu Corporation, Kyoto, Japan); 10 µL of each sample was injected on the Waters XBridge C_18_ column (250 mm × 4.6 mm, 1.7 µm, Waters Corporation, Milford, MA, USA). The temperature of the column oven was 25 °C. Mobile phase A was 0.01% (*v*/*v*) FA in H_2_0 and phase B was 0.01% (*v*/*v*) FA in ACN. The gradient of phase A was 25% from 0 to 14 min, 5% from 14 to 25 min, 20% from 25 to 25.1 min, and 25% from 25.1 to 30 min. The flow was 1 mL/min. Chromatographic parameters of detected compounds are presented in Table 3. Analytical data were analyzed using Shimadzu Browser Workstation Software For LC/MS.

### 4.4. Quantification of Photosynthetic Pigments

The endogenous level of photosynthetic pigments in *W. arrhiza* was determined using HPLC method [68]. Therefore, 0.5 g of duckweed powder was suspended in MeOH and homogenized in a bead mill (50 Hz, 10 min, TissueLyser LT; Qiagen GmbH, Düsseldorf, Germany) for cell disruption. The obtained homogenate was left in the fridge for 12 h for pigment extraction. Afterward, samples were centrifuged (2800× *g*, 10 min), and the resulting extract was analyzed.

For pigment separation and analysis, the Agilent 1260 Infinity Series HPLC apparat (Agilent Technologies, Inc., Santa Clara, CA, USA) with quaternary pump with an in-line vacuum degasser, photo-diode array detector set to monitor 350 and 700 nm, refrigerated autosampler with autoinjector sample loop, and thermostatic Eclipse XDB C_8_ column (150 mm × 4.6 mm; Agilent Technologies, Inc., Santa Clara, CA, USA) kept at 25 °C was used. The injection volume was 500 µL. The flow was 1 mL/min. The total analysis time was 40 min. Eluent A of mobile phase was MeOH/ACN/0.25 M aqueous pyridine (pH 5.0) in proportion 50/25/25 (*v*/*v*/*v*), while eluent B was MeOH/ACN/acetone in proportion 20/60/20 (*v*/*v*/*v*). The linear gradient of solvent A was as follows: from 100% in the 1st min to 60% in the 22nd min, from 60% in the 22nd min to 5% in the 38th min, from 5% in the 38th min to 100% in the 40th min. Analytical data were integrated using ChemStation software for LC systems (Agilent Technologies, Inc., Santa Clara, CA, USA).

### 4.5. Determination of Proteins’ Content

The content of soluble proteins in *W. arrhiza* was determined spectrophotometrically (Hitachi U-5100 UV-Vis spectrophotometer; Hitachi High-Tech Science Corporation, Tokyo, Japan) by the Bradford [69] method. This method is based on the ability to create ionic and hydrophobic bonds between the protein and the Coomassie Brilliant Blue G-250 dye. For the Bradford reagent preparation, 500 mg of targeted dye was dissolved in 250 mL of 95% (*v*/*v*) EtOH. Then, this mixture was filtered, and then 85% (*w*/*v*) orthophosphate acid was added. The obtained reagent was filled up with water to 1000 mL. Albumin standard was prepared through the dissolved 30 mg bovine albumin with 100 mL of distilled water. For the analytic sample preparation, the harvested and filtered duckweed was extracted in the ¼ dilution of the Bradford reagent. The blind sample was distilled water with Bradford reagent, while the standard sample was albumin with Bradford reagent. Finally, 3 mL of distilled water was added to all samples. The measurement of absorbance was performed at 595 nm 60 min after sample preparation.

### 4.6. Determination of Monosaccharides’ Content

The monosaccharides’ content was estimated spectrophotometrically (Hitachi U-5100 UV-Vis spectrophotometer; Hitachi High-Tech Science Corporation, Tokyo, Japan) according to the Somogyi-Nelson method [70,71]. A sample of *W. arrhiza* (0.5 g) was extracted in 5 mL of 62.5% (*v*/*v*) MeOH in a water bath for 30 min at 60 °C [72]. The standard sample was glucose, which was prepared by the dissolving of 30 mg glucose with 62.5% (*v*/*v*) MeOH. All samples (0.5 mL) were treated with 0.5 mL of copper reagent and placed in a boiling water bath for 20 min. Next, the 0.5 mL of arsenomolybdate reagent was added, then, after 5 min, the extract was diluted in 3.5 mL of water and mixed. The value of absorbance was read at 540 nm.

### 4.7. Statistical Treatment

The R software was used to perform statistical analyses [73]. Basic descriptive statistics were calculated for the data dataset grouped by treatment (*n* = 5) using ‘summaryBy’ function from ‘doBy’ package [74]. Then, the dataset was subjected to the one-way ANOVA (Table S1) (‘aov’ function from ‘stats’ package) followed by Tukey’s post hoc test (‘LTukey’ function from ‘laercio’ package [75]). Differences were considered significant for *p* < 0.05. The Shapiro–Wilk normality test (‘shapiro.test’ function from ‘stats’ package) and Bartlett’s test of homogeneity of variances (‘bartlett.test’ function from ‘stats’ package) were used to verify ANOVA assumptions with α = 0.05. Results from ANOVA were visualized as plots (with the help of ‘ggplot2′ package [76]) and tables.

## 5. Conclusions

The obtained results of performed experiments showed that the application of BL and/or Brz effects on the *W. arrhiza* cultures in a concentration-dependent manner. Treatment with BL in concentrations of 0.01 µM and 0.1 µM caused statistically significant increases in the growth rate, as well as the content of BRs, photosynthetic pigments, soluble proteins, and monosaccharides in duckweed after 7 days of cultivation. Otherwise, the influence of 0.001 µM BL was statistically insignificant, while the exposition on the 1 µM BL had a statistically important effect depending on the analyzed parameter. Additionally, the inhibitory effect of Brz (1 µM and 10 µM) on the growth rate and level of BRs as well as primary metabolites was verified. However, the inhibitory effect of Brz was effectively mitigated by the application of 0.1 µM BL. The level of nine BRs (BL, EBL, HBL, norBL, CT, CS, ECS, TY, and 6dTY) was reported for the first time in duckweed treated with BL and/or Brz. Additionally, the presence of EBL in *W. arrhiza* was noted for the first time.

## Figures and Tables

**Table 1:** The endogenous level (ng/g fresh weight) of Brz and BRs in *W. arrhiza* treated with Brz and/or BL after 7 days of cultivation. Data present the mean (*n* = 5) ± standard deviation. The range in square brackets corresponds to the 95% confidence interval of the mean. Means with the same letters are not significantly different (*p* ≥ 0.05) according to Tukey’s post hoc test.

Treatment	Brz	BL	EBL	HBL	norBL
Control	0	1.79 ± 0.31 ^d^	0.17 ± 0.04 ^d^	0.63 ± 0.15 ^c, d^	0.12 ± 0.04 ^c, d^
		[1.52–2.06]	[0.13–0.21]	[0.5–0.76]	[0.09–0.15]
0.1 µM Brz	911.04 ± 81.21 ^c^	1.19 ± 0.38 ^d^	0.12 ± 0.03 ^d^	0.49 ± 0.08 ^d, e^	0.09 ± 0.02 ^c, d^
	[839.86–982.22]	[0.85–1.52]	[0.1–0.14]	[0.42–0.56]	[0.07–0.11]
1 µM Brz	6987.57 ± 263.03 ^b^	0.44 ± 0.09 ^d^	0.06 ± 0.02 ^d^	0.13 ± 0.04 ^e^	0.05 ± 0.01 ^c, d^
	[6757.01−7218.13]	[0.36–0.51]	[0.05–0.08]	[0.1–0.17]	[0.04–0.06]
10 µM Brz	9381.43 ± 1007.15 ^a^	0.13 ± 0.04 ^d^	0.02 ± 0.02 ^d^	0.06 ± 0.02 ^e^	0.02 ± 0.01 ^d^
	[8498.62–10264.23]	[0.1–0.17]	[0–0.04]	[0.05–0.07]	[0.01–0.02]
0.001 µM BL	0	2 ± 0.23 ^d^	0.21 ± 0.06 ^d^	0.68 ± 0.14 ^c, d^	0.16 ± 0.04 ^c^
		[1.8–2.2]	[0.16–0.26]	[0.56–0.81]	[0.12–0.2]
0.01 µM BL	0	94.27 ± 19.58 ^d^	1.23 ± 0.17 ^d^	1.17 ± 0.38 ^b^	0.29 ± 0.06 ^b^
		[77.11–111.43]	[1.08–1.38]	[0.84–1.5]	[0.24–0.35]
0.1 µM BL	0	447.18 ± 40.86 ^b^	16.08 ± 3.2 ^a^	1.72 ± 0.3 ^a^	0.49 ± 0.11 ^a^
		[411.37–482.99]	[13.27–18.89]	[1.45–1.98]	[0.4–0.59]
1 µM BL	0	682.61 ± 82.16 ^a^	14.36 ± 1.73 ^a, b^	1.03 ± 0.18 ^b, c^	0.37 ± 0.08 ^b^
		[610.6–754.63]	[12.84–15.88]	[0.88–1.19]	[0.3–0.45]
0.1 µM BL + 0.1 µM Brz	719.6 ± 104.76 ^c^	433.03 ± 52.71 ^b^	13.37 ± 2.68 ^a, b^	1.21 ± 0.36 ^b^	0.34 ± 0.06 ^b^
	[627.78–811.43]	[386.83–479.24]	[11.02–15.72]	[0.9–1.53]	[0.28–0.39]
0.1 µM BL + 1 µM Brz	6303.29 ± 1051.26 ^b^	362.98 ± 100.02 ^b^	11.68 ± 1.93 ^b^	0.37 ± 0.13 ^d, e^	0.08 ± 0.01 ^c, d^
	[5381.82–7224.76]	[275.31–450.65]	[9.98–13.37]	[0.26–0.48]	[0.07–0.08]
0.1 µM BL + 10 µM Brz	10677.36 ± 2183.66 ^a^	259.68 ± 42 ^c^	6.51 ± 1.56 ^c^	0.1 ± 0.02 ^e^	0.03 ± 0.01 ^d^
	[8763.29–12591.42]	[222.87–296.5]	[5.14–7.87]	[0.08–0.12]	[0.02–0.03]
	**CT**	**CS**	**ECS**	**TY**	**6dTY**
Control	1.96 ± 0.61 ^b, c, d^	2.77 ± 0.29 ^c, d^	0.03 ± 0.01 ^d, e^	0.18 ± 0.05 ^d, e, f^	0.03 ± 0.01 ^b, c, d^
	[1.42–2.49]	[2.52–3.03]	[0.02–0.03]	[0.14–0.22]	[0.02–0.04]
0.1 µM Brz	1.6 ± 0.15 ^c, d, e^	2.2 ± 0.27 ^d^	0.02 ± 0 ^e, f^	0.14 ± 0.03 ^e, f, g^	0.02 ± 0.01 ^d^
	[1.46–1.73]	[1.96–2.43]	[0.01–0.02]	[0.12–0.16]	[0.01–0.03]
1 µM Brz	0.94 ± 0.16 ^d, e, f^	1.12 ± 0.11 ^e, f^	0.01 ± 0 ^f^	0.06 ± 0.01 ^f, g^	0
	[0.8–1.08]	[1.02–1.22]	[0.01–0.01]	[0.05–0.07]	
10 µM Brz	0.52 ± 0.13 ^f^	0.73 ± 0.16 ^f^	0.01 ± 0.01 ^f^	0.02 ± 0 ^g^	0
	[0.4–0.63]	[0.59–0.87]	[0–0.01]	[0.02–0.03]	
0.001 µM BL	2.08 ± 0.15 ^b, c^	2.95 ± 0.17 ^b, c^	0.03 ± 0.01 ^c, d^	0.31 ± 0.05 ^c, d^	0.04 ± 0.01 ^b^
	[1.94–2.21]	[2.8–3.1]	[0.03–0.04]	[0.27–0.35]	[0.04–0.04]
0.01 µM BL	2.76 ± 0.52 ^a, b^	3.53 ± 0.31 ^a, b^	0.04 ± 0.01 ^b, c^	0.49 ± 0.1 ^a, b^	0.05 ± 0.01 ^b^
	[2.31–3.22]	[3.26–3.8]	[0.04–0.05]	[0.4–0.58]	[0.04–0.05]
0.1 µM BL	3.47 ± 0.56 ^a^	4.02 ± 0.26 ^a^	0.05 ± 0.01 ^a^	0.57 ± 0.12 ^a^	0.06 ± 0.01 ^a^
	[2.98–3.96]	[3.79–4.24]	[0.05–0.06]	[0.47–0.67]	[0.05–0.07]
1 µM BL	2.03 ± 0.8 ^b, c^	2.75 ± 0.13 ^c, d^	0.03 ± 0 ^d, e^	0.42 ± 0.08 ^b, c^	0.04 ± 0.01 ^b^
	[1.33–2.74]	[2.63–2.87]	[0.03–0.03]	[0.35–0.48]	[0.03–0.05]
0.1 µM BL + 0.1 µM Brz	3.01 ± 1.02 ^a, b^	3.54 ± 0.44 ^a^	0.05 ± 0 ^a, b^	0.25 ± 0.14 ^d, e^	0.04 ± 0.01 ^b, c^
	[2.12–3.9]	[3.15–3.92]	[0.04–0.05]	[0.14–0.37]	[0.03–0.04]
0.1 µM BL + 1 µM Brz	1.25 ± 0.13 ^c, d, e, f^	1.6 ± 0.44 ^e^	0.01 ± 0 ^f^	0.03 ± 0.01 ^g^	0.02 ± 0.01 ^c, d^
	[1.14–1.37]	[1.21–1.99]	[0.01–0.02]	[0.02–0.04]	[0.02–0.03]
0.1 µM BL + 10 µM Brz	0.7 ± 0.11 ^e, f^	0.87 ± 0.13 ^f^	0.01 ± 0 ^f^	0	0
	[0.61–0.79]	[0.76–0.98]	[0.01–0.02]		

**Table 2:** The endogenous level of chlorophylls, carotenes, and xanthophylls (µg/g fresh weight) in *W. arrhiza* treated with Brz and/or BL after 7 days of cultivation. Data present the mean (*n* = 5) ± standard deviation. The range in square brackets corresponds to the 95% confidence interval of the mean. Means with the same letters are not significantly different (*p* ≥ 0.05) according to Tukey’s post hoc test.

Treatment	Chlorophyll *a*	Chlorophyll *b*	α-Carotene	β-Carotene	Neoxanthin
Control	162.16 ± 2.69 ^e, f^	42.19 ± 2.2 ^d, e^	1.29 ± 0.01 ^e^	1.78 ± 0.07 ^c^	0.92 ± 0.04 ^e^
	[159.8–164.51]	[40.26–44.13]	[1.28–1.3]	[1.72–1.84]	[0.88–0.95]
0.1 µM Brz	157.7 ± 1.53 ^f^	40.63 ± 1.68 ^e^	1.17 ± 0.01 ^f^	1.52 ± 0.07 ^d^	0.85 ± 0.01 ^e^
	[156.36–159.04]	[39.15–42.1]	[1.15–1.18]	[1.47–1.58]	[0.84–0.86]
1 µM Brz	120.71 ± 1.52 ^h^	29.77 ± 1.01 ^g^	0.91 ± 0.05 ^g^	1.2 ± 0.05 ^e^	0.52 ± 0.04 ^g^
	[119.38–122.04]	[28.88–30.65]	[0.87–0.95]	[1.16–1.25]	[0.48–0.55]
10 µM Brz	87.77 ± 2.41 ^i^	20.35 ± 0.89 ^i^	0.61 ± 0.02 ^h^	0.78 ± 0.1 ^g^	0.35 ± 0.02 ^h^
	[85.66–89.89]	[19.57–21.13]	[0.59–0.63]	[0.69–0.86]	[0.33–0.36]
0.001 µM BL	163.83 ± 2.73 ^e^	44.64 ± 3.02 ^d^	1.49 ± 0.03 ^d^	1.83 ± 0.03 ^c^	1.14 ± 0.08 ^d^
	[161.44–166.22]	[41.99–47.28]	[1.46–1.52]	[1.8–1.86]	[1.07–1.2]
0.01 µM BL	192.25 ± 2.52 ^c^	50.91 ± 2.27 ^c^	1.73 ± 0.06 ^c^	2.24 ± 0.08 ^b^	1.31 ± 0.02 ^b^
	[190.04–194.45]	[48.92–52.9]	[1.68–1.78]	[2.17–2.31]	[1.29–1.33]
0.1 µM BL	222.8 ± 1.73 ^a^	61.64 ± 1.23 ^a^	2.4 ± 0.05 ^a^	2.79 ± 0.12 ^a^	1.59 ± 0.04 ^a^
	[221.28–224.32]	[60.57–62.72]	[2.36–2.44]	[2.69–2.9]	[1.56–1.62]
1 µM BL	172.23 ± 2.69 ^d^	49.07 ± 1.33 ^c^	1.74 ± 0.07 ^c^	2.29 ± 0.06 ^b^	1.22 ± 0.04 ^c^
	[169.87–174.59]	[47.9–50.23]	[1.68–1.8]	[2.24–2.34]	[1.18–1.25]
0.1 µM BL + 0.1 µM Brz	202.95 ± 2.26 ^b^	55.45 ± 1.14 ^b^	2.12 ± 0.04 ^b^	2.68 ± 0.07 ^a^	1.27 ± 0.03 ^b, c^
	[200.97–204.93]	[54.46–56.45]	[2.09–2.15]	[2.62–2.74]	[1.25–1.3]
0.1 µM BL + 1 µM Brz	152.32 ± 1.44 ^g^	35.01 ± 1.7 ^f^	1.38 ± 0.04 ^e^	1.7 ± 0.01 ^c^	0.73 ± 0.01 ^f^
	[151.06–153.58]	[33.52–36.5]	[1.34–1.42]	[1.69–1.71]	[0.72–0.74]
0.1 µM BL + 10 µM Brz	122.16 ± 4.24 ^h^	25.23 ± 1.05 ^h^	0.81 ± 0.09 ^g^	1.04 ± 0.04 ^f^	0.45 ± 0.01 ^g^
	[118.44–125.88]	[24.31–26.15]	[0.72–0.89]	[1.01–1.08]	[0.44–0.46]
	**Violaxanthin**	**Astaxanthin**	**Zeaxanthin**	**Cryptoxanthin**	**Lutein**
Control	0.7 ± 0.01 ^f^	0.35 ± 0.02 ^d, e^	3.21 ± 0.06 ^d^	4.24 ± 0.08 ^e^	0.47 ± 0.01 ^d^
	[0.69–0.71]	[0.33–0.37]	[3.16–3.26]	[4.17–4.31]	[0.47–0.48]
0.1 µM Brz	0.6 ± 0.01 ^h^	0.32 ± 0.01 ^e, f^	2.73 ± 0.1 ^e^	3.96 ± 0.15 ^f^	0.42 ± 0.02 ^e^
	[0.6–0.61]	[0.31–0.33]	[2.64–2.82]	[3.83–4.09]	[0.4–0.44]
1 µM Brz	0.45 ± 0.01 ^i^	0.18 ± 0.01 ^g^	2.09 ± 0.14 ^f^	2.58 ± 0.1 ^g^	0.33 ± 0.02 ^f^
	[0.45–0.46]	[0.17–0.19]	[1.98–2.21]	[2.49–2.66]	[0.31–0.35]
10 µM Brz	0.25 ± 0.01 ^k^	0.08 ± 0.01 ^h^	0.94 ± 0.07 ^h^	2.15 ± 0.07 ^h^	0.25 ± 0.02 ^h^
	[0.25–0.26]	[0.07–0.09]	[0.88–0.99]	[2.09–2.21]	[0.22–0.27]
0.001 µM BL	0.73 ± 0.01 ^e^	0.38 ± 0.01 ^d^	3.27 ± 0.06 ^d^	4.42 ± 0.13 ^e^	0.51 ± 0.01 ^c^
	[0.72–0.74]	[0.38–0.39]	[3.22–3.32]	[4.31–4.54]	[0.5–0.53]
0.01 µM BL	0.84 ± 0.01 ^c^	0.51 ± 0.01 ^b^	3.77 ± 0.08 ^c^	5.21 ± 0.13 ^c^	0.61 ± 0.01 ^b^
	[0.83–0.85]	[0.5–0.52]	[3.69–3.84]	[5.09–5.32]	[0.6–0.62]
0.1 µM BL	0.96 ± 0.01 ^a^	0.58 ± 0.01 ^a^	4.61 ± 0.09 ^a^	6.25 ± 0.08 ^a^	0.72 ± 0.01 ^a^
	[0.95–0.97]	[0.58–0.59]	[4.53–4.69]	[6.19–6.32]	[0.71–0.73]
1 µM BL	0.78 ± 0.01 ^d^	0.44 ± 0.01 ^c^	3.65 ± 0.06 ^c^	4.93 ± 0.16 ^d^	0.6 ± 0.01 ^b^
	[0.76–0.79]	[0.43–0.45]	[3.59–3.7]	[4.79–5.07]	[0.59–0.61]
0.1 µM BL + 0.1 µM Brz	0.91 ± 0.01 ^b^	0.55 ± 0.01 ^a, b^	4.23 ± 0.08 ^b^	5.92 ± 0.05 ^b^	0.62 ± 0.01 ^b^
	[0.91–0.92]	[0.54–0.55]	[4.16–4.29]	[5.87–5.97]	[0.6–0.63]
0.1 µM BL + 1 µM Brz	0.65 ± 0.01 ^g^	0.28 ± 0.03 ^f^	2.72 ± 0.11 ^e^	3.73 ± 0.12 ^f^	0.47 ± 0.01 ^d^
	[0.64–0.66]	[0.26–0.31]	[2.62–2.82]	[3.62–3.83]	[0.46–0.48]
0.1 µM BL + 10 µM Brz	0.33 ± 0.01 ^j^	0.16 ± 0.04 ^g^	1.3 ± 0.08 ^g^	2.68 ± 0.16 ^g^	0.3 ± 0.01 ^g^
	[0.32–0.34]	[0.13–0.19]	[1.23–1.37]	[2.54–2.82]	[0.28–0.31]

**Table 3:** Chromatographic properties of Brz and BRs in positive ionization scan mode of ESI-LC-MS.

Compound	Precursor *m*/*z*	Product *m*/*z*	CE	Retention Time (min)
Brz	327.8	70.05	−25	3.976
BL	610.0	176.05	−43	11.201
EBL	610.0	190.2	−42	10.720
HBL	624.0	190.1	−43	13.118
norBL	596.0	190.0	−44	9.350
CT	433.0	415.2	−21	1.796
CS	594.1	175.95	−36	13.254
ECS	594.1	175.95	−38	13.552
6dCS	580.1	562.2	−40	23.778
TY	578.1	560.15	−37	18765
6dTY	564.2	176.15	−55	18.957

## Data Availability

Data is contained within the current article and Supplementary Materials.

## Supplementary Materials

The following are available online at https://www.mdpi.com/article/10.3390/plants10071311/s1, Table S1: One-way ANOVA results.
