# Supplementary material for: Brassinolide Enhances the Level of Brassinosteroids, Protein, Pigments, and Monosaccharides in Wolffia arrhiza Treated with Brassinazole
Source: Plants (Basel). 2021 Jun 28;10(7):1311. doi: 10.3390/plants10071311 (PMC8309140; doi:10.3390/plants10071311)
Supplement: Supplementary file 1 [file plants-10-01311-s001.zip › plants-1270331-supplementary.pdf]

Table S1. One-way ANOVA results

	Degrees of freedom	Sum of squares	Mean squares	F-statistics	*p*-value
**Biomass**					
Treatment	10	5.3099	0.53099	315.72	< 2.2e-16
Residuals	44	0.0740	0.00168	—	—
**Protein**					
Treatment	10	1608.06	160.806	163.81	< 2.2e-16
Residuals	44	43.19	0.982	—	—
**Monosaccharides**					
Treatment	10	18.523	1.85229	216.18	< 2.2e-16
Residuals	44			—	—
**Brz**					
Treatment	10	439929130	87985826	75.691	6.738e-14
Residuals	44	27898507	1162438	—	—
**BL**					
Treatment	10	2933764	293376	138.21	< 2.2e-16
Residuals	44	93398	2123	—	—
**EBL**					
Treatment	10	2269.21	226.921	93.704	< 2.2e-16
Residuals	44	106.55	2.422	—	—
**HBL**					
Treatment	10	14.3743	1.43743	34.295	< 2.2e-16
Residuals	44	1.8442	0.04191	—	—
**norBL**					
Treatment	10	1.32676	0.132676	46.687	< 2.2e-16
Residuals	44	0.12504	0.002842	—	—
**CT**					
Treatment	10	46.243	4.6243	18.494	1.159e-12
Residuals	44	11.002	0.2500	—	—
**CS**					
Treatment	10	65.921	6.5921	90.15	< 2.2e-16
Residuals	44	3.217	0.0731	—	—
**ECS**					
Treatment	10	0.0116151	0.00116151	42.56	< 2.2e-16
Residuals	44	0.0012008	0.00002729	—	—
**TY**					
Treatment	10	1.99026	0.199026	41.044	< 2.2e-16
Residuals	44	0.21336	0.004849	—	—
**6dTY**					
Treatment	10	0.0228112	0.00228112	40.348	< 2.2e-16
Residuals	44	0.0024876	0.00005654	—	—
**Chlorophyll *a***					
Treatment	10	76266	7626.6	1254.2	< 2.2e-16
Residuals	44	268	6.1	—	—

**Chlorophyll *b***					
Treatment	10	8245.1	824.51	280.56	< 2.2e-16
Residuals	44	129.3	2.94	—	—
**$\alpha-$carotene**					
Treatment	10	15.1611	1.51611	622.02	< 2.2e-16
Residuals	44	0.1072	0.00244	—	—
**$\beta-$carotene**					
Treatment	10	21.3051	2.13051	430.27	< 2.2e-16
Residuals	44	0.2179	0.00495	—	—
**Neoxanthin**					
Treatment	10	8.0416	0.80416	631.81	< 2.2e-16
Residuals	44	0.0560	0.00127	—	—
**Violaxanthin**					
Treatment	10	2.63503	0.263503	2840.9	< 2.2e-16
Residuals	44	0.00408	0.000093	—	—
**Astaxanthin**					
Treatment	10	1.34940	0.13494	421.95	< 2.2e-16
Residuals	44	0.01407	0.00032	—	—
**Zeaxanthin**					
Treatment	10	66.487	6.6487	863.94	< 2.2e-16
Residuals	44	0.339	0.0077	—	—
**Cryptoxanthin**					
Treatment	10	90.946	9.0946	661.55	< 2.2e-16
Residuals	44	0.605	0.0137	—	—
**Lutein**					
Treatment	10	1.09665	0.109665	420.13	< 2.2e-16
Residuals	44	0.01149	0.000261	—	—
